# Supplementary material for: Climatic windows for human migration out of Africa in the past 300,000 years
Source: Nat Commun. 2021 Aug 24;12:4889. doi: 10.1038/s41467-021-24779-1 (PMC8384873; doi:10.1038/s41467-021-24779-1)
Supplement: Supplementary file 1 — Supplementary Information [file 41467_2021_24779_MOESM1_ESM.pdf]

Supplementary Information

Climatic windows for human migration out of Africa in the past 300,000 years

Robert M. Beyer, Mario Krapp, Anders Eriksson, Andrea Manica

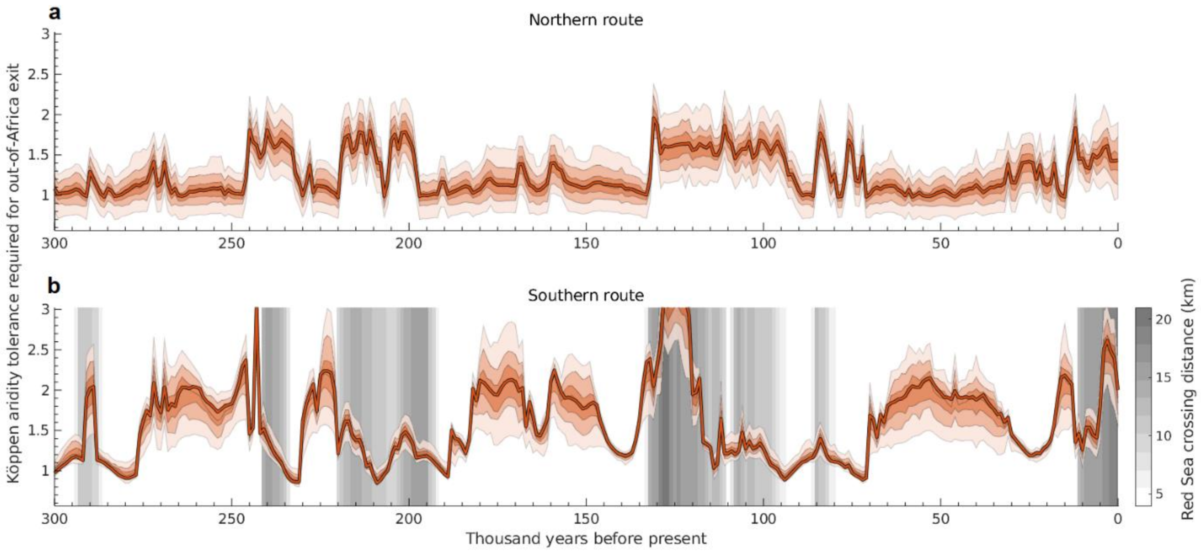

Supplementary Figure 1. Results equivalent to Fig. 1 but based on Köppen aridity instead of precipitation. (a) Tolerance to low Köppen aridity that would have been required for *Homo sapiens* to leave the African continent in the past 300k years. Orange lines represent minimum aridity levels for which a connected path between Africa and Eurasia along the (a) northern (b) southern route existed, i.e., the minimum levels that humans would have had to withstand for a successful exit along these routes, based on climatological normals at 1k year time steps (Methods). Thus, higher values correspond to a more favourable climate along the routes. Orange shades represent the 10th–90th percentiles of the minimum aridity tolerance required for a successful exit based on decadal scale climate (Methods). Grey shades in (b) represent the minimum distance needed to cover on water to reach the Arabian Peninsula from Africa.

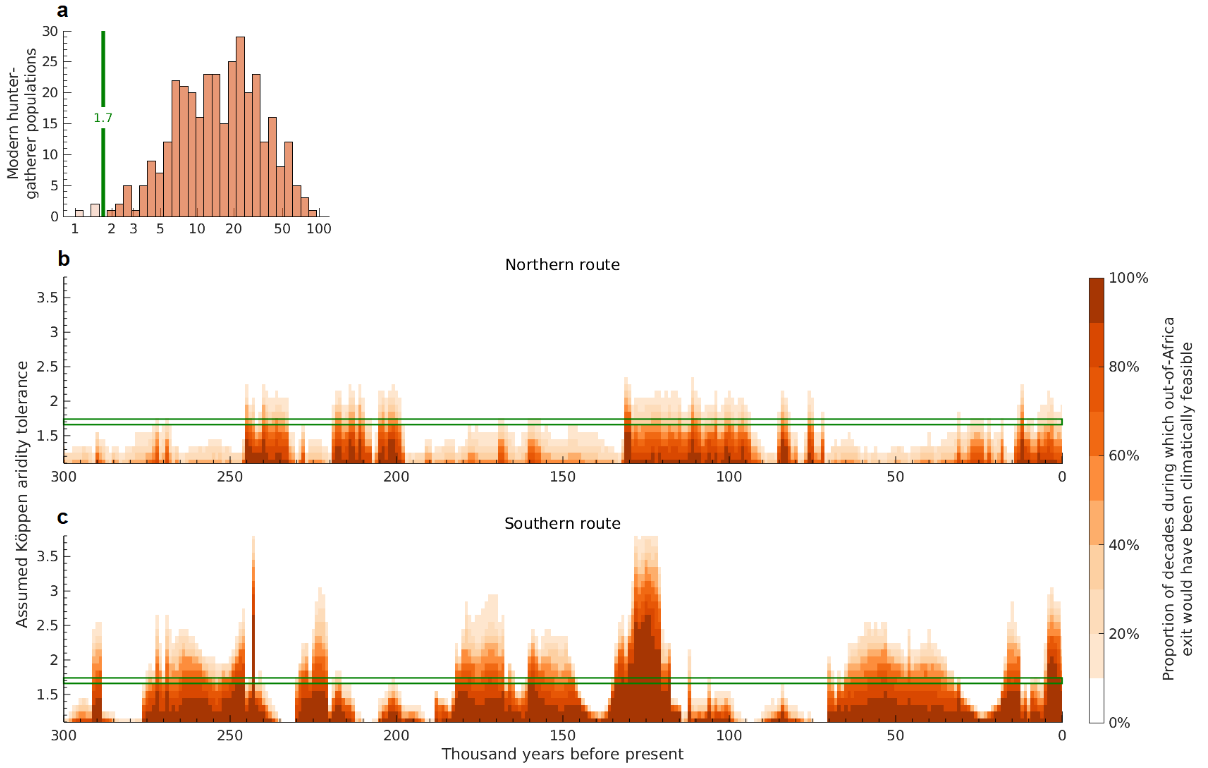

Supplementary Figure 2. Results equivalent to Fig. 2 but based on Köppen aridity instead of precipitation. (a) Distribution of modern hunter-gatherer populations under different aridity levels. Transparent bins correspond to populations located in close vicinity of a water source, which are not considered to be constrained by aridity. (b),(c) Percentage of decades within a given millennium (x-axis) during which a connected path between Africa and Eurasia along the (b) northern and (c) southern route existed for a given threshold tolerance to low aridity (y-axis). The green band represents our empirically estimated threshold of 1.7.
