# Supplementary material for: Climatic windows for human migration out of Africa in the past 300,000 years
Source: Nat Commun. 2021 Aug 24;12:4889. doi: 10.1038/s41467-021-24779-1 (PMC8384873; doi:10.1038/s41467-021-24779-1)
Supplement: Supplementary file 3 — Description of Additional Supplementary Files [file 41467_2021_24779_MOESM3_ESM.pdf]

Description of Additional Supplementary Files

Supplementary Movie 1. Maps of annual precipitation $\overline{\mathbf{P}}_{\sim 0.5°}(t)$ (Eq. (2)), and mean annual temperature $\overline{\mathbf{T}}_{\sim 0.5°}(t)$ (Eq. (5)), at 1k year time steps between 300k years ago and the present.

Supplementary Movie 2. Maps of the percentage of decades during which grid cells were inhabitable based on precipitation (cf. Fig. 3a–e) and aridity, at 1k year time steps between 300k years ago and the present.
